# Estimation of Stature from Arm Span, Arm Length and Tibial Length among Adolescents of Aged 15–18 in Addis Ababa, Ethiopia

**DOI:** 10.4314/ejhs.v31i5.18

**Published:** 2021-09

**Authors:** Abay Mulu, Bereket Sisay

**Affiliations:** 1 Department of Anatomy, College of Health Sciences, Addis Ababa University, Addis Ababa, Ethiopia; 2 Biomedical Sciences, College of Health Sciences, Yirgalem Hospital Medical College, Yirgalem, Ethiopia

**Keywords:** Stature, Arm span, Arm length, Tibial length, Estimation

## Abstract

**Background:**

Knowing the relationship between stature and different anatomical anthropometric parameters help forensic scientists, anatomists and clinicians to estimate standing height from mutilated remains of body parts in clinical practices and forensic investigations. It is a necessity when measuring height is unenviable due to certain medical conditions and in field studies. This study aims to estimate stature from arm span, arm length and tibial length among adolescents of age 15–18 in Ethiopia.

**Methods:**

A school based cross-sectional study was carried out among 416 high school students in Addis Ababa, Ethiopia from May to June 2019. Stratified multi-stage sampling techniques were used to select the study participants. Anthropometric measurement including weight, height, arm span, arm length and tibial length was measured. Data entry was done by Epi-Data a version 4.4.3.1 and data analysis was carried out by Statistical Package for Social Sciences version 23. Regression models and multiplication factors were generated for estimation of height from anthropometric parameters.

**Result:**

From total participants 51.4% were females and 48.6% were males. The mean height of study participants was 164.36±8.89cm for males and 155.75±5.86cm for females. The correlation coefficients(R) of anatomical anthropometric measurements with height were: arm span (males R=0.843, females R=0.708), arm length (males R=0.806, females R=0.635), and tibial length (males R=0.738, females R=0.611).

**Conclusion:**

Stature predicted from arm span, arm length, and tibial length is a valid indicator of height. Arm span was appeared to be the best predictor of stature.

## Introduction

Stature is the highest distance measured from the point where the heel touches the floor to the vertex of the head while the person is erect (1). Anthropometry, which deals with describing human form in numbers, has been widely used in forensic investigations and in many clinical circumstances. Sex, age, stature and race will help to nail down the pool of victims match during forensic investigations. From all of the above variables stature is the most vital tool for personal identification in case when only remains of a human body found (2).

Even though standing height plays a big role in personal identification of an individual in case of medico-legal scenario and in many clinical conditions, there are conditions which hinder the direct measurement of standing height like spinal and limb deformities, amputations, fractures, scoliosis, paralysis and pain, in this cases segment of the body are used for estimating the standing height of an individual (3).

Moreover, problems and difficulties in measurement of height can be encountered in field studies compared with clinical settings because of the portability, accessibility, and expense of specialized equipment used for measurement of height. Therefore, surrogate measures of height that can be obtained by simple and portable tools are mandatory. Therefore, most regression formulas derived are meant to be specific for a particular region and can be used only in that particular area of study and for that particular age group in which the study was conducted. It is indeed, necessary to derive regression equations, which are age group, gender, ethnic, and geographic area specific (3,4). As my search has shown there is no study done in Ethiopian adolescents of age below 18 on estimation of stature from arm span, arm length and tibial length, so this study aims to estimate stature from arm span, arm length and tibial length among adolescents of age 15–18 in Addis Ababa, Ethiopia.

## Materials and Methods

**Study setting and study population**: School based descriptive cross-sectional study was carried out among 416 study participants of age 15–18 from selected public and private general secondary schools in Addis Ababa, Ethiopia. The study was conducted from May to June 2019. The sample size for the study was determined based on formula for single population proportion, using, level of significance as 95%, (Zα2=1.96), margin of error 5 and proportion of 0.5.

Multi-stage sampling method was used to select samples that fulfil inclusion criteria. A total of 15 schools, (10 non-governmental and 5 governmental) schools were selected randomly. And the study subjects were selected from selected sections by systematic random sampling technique using student list of the class.

**Data collection and instrument**: The data were collected through structured, pretested interviewer-administered questionnaire and anthropometric parameters were taken by direct measurements. According to Marfell-Jones (5), the anthropometric measurements were taken using the protocol of the International Society for the Advancement of Kinanthropometry (ISAK). Length was measured by using nonelastic tape meter.

Stature was measured using a portable stadiometer and put to the nearest point two decimal places of centimeters. Weight was also measured with standard mechanical balance and recorded to the nearest 0.1kg.

Arm span measurements were taken from the tip of the middle finger of one arm to the tip of the middle finger of the other arm (dactylion to dactylion) with the arms outstretched at right angles of 180° to the body and with extended elbow and wrist, and the palms facing directly forward.

Arm length was taken from the tip of humerus (acromion) bone to tip of the middle finger (dactylion) of right arm while arm hanging down wards lateral to the body. Tibial length was measured from the medial most superficial point on upper border of medial condyle of tibia and tip of medial malleolus of right leg.

All the anthropometric measurements except for weight were measured when the subjects were standing in the Frankfort horizontal plane.

**Data analysis and processing**: Data were edited, coded, and entered into Epi-Data version 4.4.3.1 and were exported for analyses to SPSS for windows version 23. Statistical significance was set at p<0.05. Simple and multiple linear regression models and multiplication factors were generated to estimate stature of study participants from arm span, arm length and tibial length.

Before conducting analysis of the present data several tests for basic assumptions of linear regression like tests of linearity, tests of homoscedasticity and tests of normality; and reliability analysis were taken into consideration.

Reliability of the present data was assessed by computing Cronbach's Alpha. In the present study the computed value of Cronbach's alpha was found to be 0.911 which shows that there was high internal consistency between all items in the questionnaire.

## Results

A summary of the anthropometric measurements in both sexes is shown in Table 1. The mean of the height and arm span for male subjects was 164.36±8.89cm and 165.7±9.46cm, respectively. And for female subjects it was 155.74±5.86cm and 155.96±6.95cm. Independent sample t-test was generated to assess the presence of sexual dimorphism between Height and Arm span and the result illustrates that there was statistically significant higher mean for male study subjects than their female counter parts (body height: t=11.714; p<.000, and arm span: t=12.001; p<.000).

**Table 1:** Anthropometric measurements of study subjects

Variable	Measurements	
	
	Range	Mean ± SD
**Body height**		
Male	36.5–192.0	64.36±8.89
Female	43.0–176.0	55.74±5.86
**Arm Length**		
Male	4.2–88.90	6.74±4.67
Female	3.1–82.0	2.49±3.48
**Arm Span**		
Male	31.5–186.1	65.7±9.46
Female	36.2–172.5	55.96±6.95
**Tibial Length**		
Male	2.1–47.3	0.31±2.91
Female	1.9–44.7	7.85±2.26

SD-Standard Deviation

The mean of the arm length and tibial length for male subjects was 76.74±4.67cm and 40.31±2.91cm, respectively. And for female subjects it was 72.49±3.48cm and 37.85±2.26cm. Independent sample t-test was also generated to assess the presence of sexual dimorphism between arm length and tibial length and the result illustrates that there was statistically significant higher mean for male study subjects than the females (Arm length: t=10.547; p<.000, and Tibial length: t=9.623; p<.000). Moreover, it was also observed that most 60.4% of male and 55.1% of female adolescents have higher measurement of height than arm span as shown in Table 2.

**Table 2:** Distribution of study participants on the basis of height and arm span in Addis Ababa, Ethiopia

Groups	Male	Female	Total
Height = Arm span	3(1.5%)	12(5.6%)	15(3.6%)
Height < Arm span	77(38.1%)	84(39.3%)	161(38.7%)
Height > Arm span	122(60.4%)	118(55.1%)	240(57.6%)

Total	202(100%)	214(100%)	416(100%)

**Correlation between standing height and anatomical anthropometric parameters**: The simple correlation coefficient and their 95% confidence interval analysis between the anthropometric measurements are presented in Table 3. The relationships between body height and anatomical anthropometric parameters were high and significant in study subjects regardless of sex. Arm span was found to be highly correlated with stature in both sexes. The mean value of multiplication factors for each anatomical anthropometric parameters of male and female study subjects is illustrated in Table 4.

**Table 3:** Correlation of height with anatomical anthropometric parameters of male and female participants in Addis Ababa, Ethiopia

Variable	Correlation coefficient	95% confidence interval	Significant p-value
**Arm span**			
Male	0.843	0.842–0.844	0.000
Female	0.708	0.705–0.710	0.000
**Arm length**			
Male	0.806	0.805–0.807	0.000
Female	0.635	0.630–0.638	0.000
**Tibial length**			
Male	0.738	0.734–0.740	0.000
Female	0.611	0.608–0.615	0.000

**Table 4:** Multiplication factor for each anatomical anthropometric parameter of male and female study participants in Addis Ababa, Ethiopia

Subject	Arm span MF mean ± SD	Arm length MF mean± SD	Tibial length MF mean ± SD
Male	0.992±0.30	2.143±0.077	4.086±0.201
Female	0.999±0.32	2.151±0.082	4.123±0.194

MF-Multiplication Factor, SD-Standard Deviation

**Simple linear regression equations to estimate of stature from anatomical anthropometric parameters for male and female study participants**: A summary of regression results for male and female study subjects is provided in the Table 5 below. The high values of the regression coefficient (coefficient of determination) signify that all anatomical anthropometric parameters especially that of arm span significantly predicts body height in both sexes.

**Table 5:** Estimation of stature from anatomical anthropometric parameters for male and female study participants in Addis Ababa, Ethiopia

Variable	Regression coefficient(R^2^)	SEE	Regression equation	p-value
**Arm span**				
Male	0.711	4.790	33.11+0.792(AS)	.000
Female	0.501	4.153	62.59+0.597(AS)	.000
**Arm length**				
Male	0.649	5.280	46.71+1.53(AL)	.000
Female	0.403	4.546	78.36+1.06(AL)	.000
**Tibial length**				
Male	0.545	6.012	73.53+2.25(TL)	.000
Female	0.373	4.654	95.96+1.58(TL)	.000

SEE- Standard Error of Estimate, AS- Arm Span, AL- Arm Length TL- Tibial Length

**Step wise multiple linear regression including bilateral and different anatomical anthropometric parameters**: On multivariable linear regression analyses, arm span and arm length, arm span and tibial length, tibial length and arm length, and arm span, arm length and tibial length significantly predicted standing height measurement (p<0.05) (Table 6).

**Table 6:** Step-wise multiple linear regression for estimation of stature from bilateral and different anthropometric parameters for study participants in Addis Ababa, Ethiopia

Parameter	Regression coefficient(R^2^)	SEE	Dublin Watson	Regression equation	P-value
**Male**					
AS-AL	0.726	4.670	1.924	31.05+0.56(AS)+0.50(AL)	0.000
AS-TL	0.756	4.413	1.745	28.71+0.6(AS)+0.89(TL)	0.000
AL-TL	0.710	4.815	1.995	40.22+1.04(AS)+1.06(TL)	0.000
AS-AL-TL	0.764	4.347	1.866	27.97+0.49(AS)+0.27(AL)+0.85(TL)	0.000
**Female**					
AS-AL	0.507	4.139	1.846	60.97+0.5(AS)+0.23(AL)	0.000
AS-TL	0.549	3.961	1.883	58.12+0.45(AS)+0.70(TL)	0.000
AL-TL	0.486	4.225	1.808	68.38+0.72(AL)+0.92(TL)	0.000
AS-AL-TL	0.551	3.960	1.854	56.95+0.39(AS)+0.15(AL)+0.68(TL)	0.000

SEE- Standard, Error of Estimate, AS- Arm Span, AL- Arm Length, TL- Tibial Length

## Discussion

Age and sex along with stature constitute the important parameters for establishing the identity of the remains of the human body in case of forensic investigation (6).

The mean differences of stature, arm span, arm length and tibial length among Addis Ababa male and female study subjects of the present study showed statistically significant difference (p ∼0.00). This is in line with studies like (7–9). However, some studies indicated that there is no significant mean difference of anthropometric parameters between sexes (10).

The estimation of stature using various anthropometric measurements are quite the age-old investigations over the past centuries and it has been attempted by many authors in many countries. As it is already mentioned, all of them estimated stature from various anatomical anthropometric measurements, but it is important to emphasize that the arm span has been derived the most reliable parameter for predicting body height of an individual (11).

In the study conducted by Steele and Chenier (12), the arm span was nearly 8.3cm more than the body height for black population, whereas for white population this difference was only 3.3cm (11). They have noted in their study done on South Indian females, the arm span was nearly 2.5cm higher than stature. In study (13), arm span was 5.8cm more than body height for Nigerian males whereas, for Nigerian females, this difference was only 4cm which is similar to that noted in the white population although they are black. The findings of the above studies contradict with the result of the present study where 60.4% of male and 55.1% of female study participants have higher mean value of height than arm span, while only 38.1% of male and 39.3% of females have higher arm span.

The results of many studies conducted across the globe indicated that arm span and height showed the highest correlation coefficient than any other anthropometric measurements. For example, a study (11) reported that the correlation coefficient was R=0.82, while in another study (8) correlation was R=0.87 for males and R=0.81 for the female population. In the most recent studies, (13) reported that correlation was R=0.83, while others (14) reported that the correlation was R=0.861 for males and R=0.809 for female study subjects.

The findings from the present study was found to be in line with the findings of the above study where arm span showed the highest value of correlation than any other anthropometric parameters for both sexes(R=0.843 for males and R=0.708 for females).Arm length also has shown to be strongly associated with stature in the present study with correlation coefficient(R) of 0.806 for male and 0.635 for female study subjects; this is in line with studies done by Dorjee et al. (10).

Tibial length is strongly associated with stature in the present study with correlation coefficient(R) of 0.738 for male and 0.611 for female study subjects, this is also in line with numerous studies (10,15,16).

**Choosing the best regression equation for stature estimation**: The study of many researchers (17–20) reported that the best stature predictor to be arm span followed by those models containing arm length and then tibial length. In the present study it was evident that simple linear regression equations derived from arm span was found to be the best predictor of standing height with the largest value of regression coefficient R^2^-0.711 for male and R^2^-0.501 for female study participants, followed by regression equations derived from arm length and tibial length respectively.

However, it was evident that in the present study step wise multiple linear regression equation consisting arm span, arm length and tibial length were found to be the best predictor of stature than any other equation consisting one or two parameters in both sexes. This is in line with numerous studies conducted in different areas and in different study set up (2,21,4).

In the present study height prediction equation using arm span, a centimeter increase in arm span increased height by 0.792cm in male and 0.597cm in female study subjects with p<0.001. Thus, height can be predicted from arm span as: Height = 33.11+0.792 (Arm span) in male and Height = 62.59+0.597 (Arm span) in female.

The present study also evaluates accuracy of estimated stature by comparing the mean values of estimated and actual stature and dependent student t- test was performed to justify the existence of mean difference for both sexes separately. The mean value of height estimated from arm span, arm length, and tibial length show statistically insignificant difference (p>0.05) when compared with mean value of stature of study participants. The findings of the present study is in agreement with numerous studies like (22,23,4) where student t-test was computed and the results indicate that there is no statistically significant mean difference between actual and estimated heights.

In conclusion, stature has showed statistically significant correlation with arm span, arm length and tibial length in both sexes. Arm span highly correlates with height and regression equations fitted from arm span was found to be best estimator of height. The strength of prediction of regression equations in general increased with increasing number of anatomical anthropometric parameter and from linear to stepwise multiple regressions. There was no statistically significant mean difference between estimated and actual stature in both male and female study participants.

## References

[1] Datta Banik S (2011). Arm span as a proxy measure for height and estimation of nutritional status: a study among Dhimals of Darjeeling in West Bengal India. Ann Hum Biol.

[2] Patel PN, Tanna J, Kalele SD (2012). Correlation between Hand Length and Various Anthropometric Parameters. International journal of medical toxicology and forensic medicine.

[3] Quanjer PH, Capderou A, Mazicioglu MM, Aggarwal AN, Banik SD, Popovic S, Tayie FA, Golshan M, Ip MS, Zelter M (2014). All-age relationship between arm span and height in different ethnic groups. EurRespir J.

[4] Digssie A, Argaw A, Belachew T (2018). Developing an equation for estimating body height from linear body measurements of Ethiopian adults. J PhysiolAnthropol.

[5] Marfell-Jones M, Olds T, Stewart A, Carter L (2006). International Standards for Anthropometric Assessment.

[6] Rastogi P, Kanchan T, Menezes RG, Yoganarasimha K (2009). Middle finger length--a predictor of stature in the Indian population. Med Sci Law.

[7] Yabanci N, Kiliç S, Simşek I (2010). The relationship between height and arm span, mid-upper arm and waist circumferences in children. Ann Hum Biol.

[8] Zverev YP (2003). Relationship between arm span and stature in Malawian adults. Ann Hum Biol.

[9] Ibegbu A, David ET, Hamman WO, Umana UE, Musa SA (2014). Height determination using hand length in Nigerian school children. J Morphol Sci.

[10] Dorjee B, Sen J (2016). Estimation of stature from arm span, arm length and tibial length among Bengalee. journal of human biological review.

[11] Mohanty SP, Babu SS, Nair NS (2001). The use of arm span as a predictor of height: A study of South Indian women. J Orthop Surg.

[12] Steele MF, Chenier TC (1990). Arm-span, height, and age in black and white women. Ann Hum Biol.

[13] Goon DT, Toriola AL, Musa DI, Akusu S (2011). The relationship between arm span and stature in Nigerian adults. Kinesiology.

[14] Bjelica D, Popovic S, Kezunovic M, Petkovic J, Jurak G, Grasgruber P (2012). Body height and its estimation utilising arm span measurements in Montenegrin adults. Anthropological Notebooks.

[15] Mahakkanukrauh P, Khanpetch P, Prasitwattanseree S, Vichairat K, Troy Case D (2011). Stature estimation from long bone lengths in a Thai population. Forensic Sci Int.

[16] Duyar I, Pelin C (2003). Body height estimation based on tibia length in different stature groups. Am J Phys Anthropol.

[17] de Lucia E, Lemma F, Tesfaye F, Demisse T, Ismail S (2002). The use of arm span measurement to assess the nutritional status of adults in four Ethiopian ethnic groups. Eur J Clin Nutr.

[18] Yousafzai AK, Filteau SM, Wirz SL, Cole TJ (2003). Comparison of armspan, arm length and tibia length as predictors of actual height of disabled and nondisabled children in Dharavi, Mumbai, India. Eur J Clin Nutr.

[19] Neyestani TR, Dad-Khah M, Haidari H, Zowghi T, Maddah M, Nematy M, Aliabadi M (2011). Determination of the actual height predictors in Iranian healthy children. Acta Med Iran.

[20] Sah R, Kumar A, Bhaskar R (2013). Body height and its estimation utilizing arm span measurements in population of Birgunj Area of Nepal: An Anthropometric study. Journal of College of Medical Sciences-Nepal.

[21] Popovic S, Bjelica D, Tanase GD, Milasinovic R (2015). Body height and its estimation utilizing arm span measurements in Bosnian and Herzegovinian adults. Monten J Sports Sci Med.

[22] Hossain S, Begum J, Akhter Z (2011). Measurement of Stature from Arm-span. An Anthropometric Study on Garo Tribal Bangladeshi Females. Bangladesh Journal of Anatomy.

[23] Kamal R, Yadav P (2016). Estimation of stature from different anthropometric measurements in Kori population of North India. Egyptian Journal of Forensic Sciences.

